# Resignation

**Published:** 1884-09-20

**Authors:** 


### EDITORIALS AND MISCELLANEOUS.

### RESIGNATION.

Dr. Thad. Johnson has resigned the chair of Surgery in the SOUTHERN MEDICAL COLLEGE, Atlanta, having decided to move to California. The Doctor goes to the West for the benefit of his health. We wish him abundant success in his new field of labor.

We are gratified to state to the friends of the Institution that the Chair made vacant by Dr. Johnson's resignation has been well and ably filled by the appointment of Dr. J. McF. Gaston, formerly of Columbia, S. C.

Doctor Gaston is a gentleman of fine attainments in the Profession. He is an able writer and an active and progressive man, as evinced by his numerous contributions to the medical journals. He distinguished himself as a surgeon in the Confederate army, and his fame as an operative surgeon has followed him from South Carolina, where he formerly resided, and, also, from Brazil, to which country he repaired for a time after the war.

THE SOUTHERN MEDICAL COLLEGE is fortunate in securing the services of such a man.

